# Supplementary material for: Discharge Interventions for First Nations People with Injury or Chronic Conditions: A Protocol for a Systematic Review
Source: Int J Environ Res Public Health. 2022 Sep 8;19(18):11301. doi: 10.3390/ijerph191811301 (PMC9517407; doi:10.3390/ijerph191811301)
Supplement: Supplementary file 1 [file ijerph-19-11301-s001.zip › Supplementary File S2-planned search strategy.pdf]

SEARCH STRATEGY

PubMed:

(("indige*"[All Fields] OR "aborigin*"[All Fields] OR "Torres Strait Islander"[All Fields] OR "First People"[All Fields] OR "Inuit"[All Fields] OR "First Nation"[All Fields] OR "Maori"[All Fields] OR "Native American"[All Fields] OR "Native"[All Fields] OR "Sami"[All Fields]) AND (("clinical trial"[Publication Type] OR "journal article"[Publication Type] OR "observational study"[Publication Type] OR "review"[Publication Type] OR "systematic review"[Filter]) AND "humans"[MeSH Terms] AND ("medline"[Filter] OR "jsubsetn"[All Fields]) AND 2010/01/01:2021/02/01[Date - Publication] AND "english"[Language]) AND (("injur*"[All Fields] OR "burn*"[All Fields] OR "Chronic condition"[All Fields] OR "Chronic illness"[All Fields] OR "Chronic disease"[All Fields] OR "Chronically ill"[All Fields] OR "Long term condition"[All Fields]) AND (("clinical trial"[Publication Type] OR "journal article"[Publication Type] OR "observational study"[Publication Type] OR "review"[Publication Type] OR "systematic review"[Filter]) AND "humans"[MeSH Terms] AND ("medline"[Filter] OR "jsubsetn"[All Fields]) AND 2010/01/01:2021/02/01[Date - Publication] AND "english"[Language])) AND (("discharge plan*"[All Fields] OR ("discharges"[All Fields] OR "discharging"[All Fields] OR "Patient discharge"[MeSH Terms] OR ("patient"[All Fields] AND "discharge"[All Fields]) OR "Patient discharge"[All Fields] OR "discharge"[All Fields] OR "discharged"[All Fields]) OR "Patient discharge"[All Fields] OR "Discharge process"[All Fields] OR "Discharge education"[All Fields] OR "Discharge intervention"[All Fields] OR "Follow UP"[All Fields] OR "Aftercare"[All Fields]) AND (("clinical trial"[Publication Type] OR "journal article"[Publication Type] OR "observational study"[Publication Type] OR "review"[Publication Type] OR "systematic review"[Filter]) AND "humans"[MeSH Terms] AND ("medline"[Filter] OR "jsubsetn"[All Fields]) AND 2010/01/01:2021/02/01[Date - Publication] AND "english"[Language]))) AND ((2010/1/1:2021/2/1[pdat]) AND (english[Filter]))

CINAHL:

S1 AND S2 ANS S3 (limiters: date published :01/01/2010-01/02/2021. Language: English) **(search mode: find all my search terms)**

S1: Indige* OR Aborigin* OR indian OR “Torres Strait Islander” OR “First People” OR Inuit OR “First Nation” OR Maori OR “Native American” OR Native OR Sami **(search mode: Boolean/phrase)**

S2: Injur* OR Burns OR "Chronic condition" OR "Heart disease" OR “Chronic kidney disease” OR “Cystic fibrosis” OR Diabetes OR “COPD” OR "chronic obstructive pulmonary disease" OR "Chronic illness" OR "Chronic disease" OR "Chronically ill" OR "Long term condition"**(search mode: Boolean/phrase)**

S3: Discharge plan* OR discharge OR Patient discharge OR Discharge process OR Discharge management OR Discharge education OR Discharge intervention OR "Follow UP" OR Aftercare OR "Model of aftercare"**(search mode: Boolean/phrase)**

Embase:

1. (indige* or aborigin* or "torres strait islander" or "first nation" or "first people" or "native american" or "native" or "maori" or "sami" or "inuit").mp. [mp=title, abstract, heading word, drug trade name, original title, device manufacturer, drug manufacturer, device trade name, keyword, floating subheading word, candidate term word]

2. ("Discharge plan*" or discharge or "Patient discharge" or "Discharge process" or "Discharge management" or "Discharge education" or "Discharge intervention" or "Follow UP" or Aftercare).mp. [mp=title, abstract, heading word, drug trade name, original title, device manufacturer, drug manufacturer, device trade name, keyword, floating subheading word, candidate term word]

3. (injur* or burn* or "chronic disease" or "chronic condition" or "chronic illness" or "chronically ill" or "long term condition" or "long term disease").mp. [mp=title, abstract, heading word, drug trade name, original title, device manufacturer, drug manufacturer, device trade name, keyword, floating subheading word, candidate term word]

4. 1 and 2 and 3

5. limit 4 to (human and english language and yr="2010 - 2021")

Web of Science:

#3 AND #2 AND #1 Refined by: PUBLICATION YEARS: (2021 OR 2013 OR 2020 OR 2012 OR 2019 OR 2011 OR 2018 OR 2010 OR 2017 OR 2016 OR 2015 OR 2014)

#1 TS= (indige* OR aborigin* OR "torres strait islander" OR "first nation" OR "first people" OR "native american" OR "native" OR"maori" OR "sami" OR "inuit") Indexes=SCI-EXPANDED, SSCI, A&HCI, CPCI-S, CPCI-SSH, BKCI-S, BKCI-SSH, ESCI, CCR-EXPANDED, IC Timespan=All years

#2 TS= (Injur* OR Burns OR "Chronic condition"OR "Chronic illness" OR "Chronic disease" OR "Chronically ill" OR "Long term condition") Indexes=SCI-EXPANDED, SSCI, A&HCI, CPCI-S, CPCI-SSH, BKCI-S, BKCI-SSH, ESCI, CCR-EXPANDED, IC Timespan=All years

#3 TS= ("Discharge plan*" or discharge or "Patient discharge" or "Discharge process" or "Discharge management" or "Discharge education" or "Discharge intervention" or "Follow UP" or Aftercare) Indexes=SCI-EXPANDED, SSCI, A&HCI, CPCI-S, CPCI-SSH, BKCI-S, BKCI-SSH, ESCI, CCR-EXPANDED, IC Timespan=All years
